# CT-based habitat imaging integrated with radiomics and clinicopathology for noninvasive prediction of microvascular invasion in hepatocellular carcinoma

**DOI:** 10.3389/fonc.2026.1832615

**Published:** 2026-05-22

**Authors:** Shuangxi Chen, Xushuang Qin, Shanni Dong, Xiaoshu Zhu, Yang Liu, Jun Chen, Ruizhong Ye, Li Zhu

**Affiliations:** 1 Cancer Center, Department of Ultrasound Medicine, Zhejiang Provincial People’s Hospital, Affiliated People’s Hospital, Hangzhou Medical College, Hangzhou, Zhejiang, China; 2 Cancer Center, Department of Interventional Medicine, Zhejiang Provincial People’s Hospital, Affiliated People’s Hospital, Hangzhou Medical College, Hangzhou, Zhejiang, China

**Keywords:** CT, habitat analysis, hepatocellular carcinoma, MVI, radiomics

## Abstract

**Objective:**

To develop and validate a CT-based habitat imaging model incorporating intratumoral microenvironment heterogeneity analysis for noninvasive preoperative prediction of microvascular invasion (MVI) in hepatocellular carcinoma (HCC), addressing limitations of conventional radiomics in characterizing intra-tumor heterogeneity.

**Methods:**

This retrospective study included 216 patients with pathologically confirmed HCC undergoing resection. Preoperative portal venous phase contrast-enhanced CT and corresponding postoperative histopathologic MVI status were collected. Habitat (functional heterogeneous subregion) and conventional radiomic features were extracted. A habitat risk score and radiomic score were calculated. Clinical-pathologic factors (Edmondson grade, p53/CD10 expression, tumor diameter) identified via univariate/multivariate analyzes were integrated into a predictive model visualized as a nomogram. Performance was assessed by AUC, calibration curves, and decision curve analysis (DCA). DeLong’s test compared ROC curves.

**Results:**

In the training set, the combined model achieved an AUC of 0.862 (95% CI: 0.797–0.926); in the validation set, AUC was 0.814 (95% CI: 0.710–0.918), both significantly outperforming individual models (all P < 0.05). Calibration curves showed good agreement (Hosmer–Lemeshow P = 0.60). DCA indicated net benefit at thresholds of 15%–65%. DeLong’s test confirmed the combined model had higher AUC than the clinical model (Z = –3.21, P < .05) and radiomics-only model (Z = –2.05, P < .05).

**Conclusion:**

The CT-based habitat imaging model quantified intratumoral heterogeneity and, combined with clinicopathologic data, provided a reliable noninvasive tool for preoperative MVI risk stratification in HCC, with strong clinical potential.

## Introduction

Hepatocellular carcinoma (HCC) is one of the leading causes of cancer-related mortality worldwide (1, 2), and its aggressive progression is frequently associated with microvascular invasion (MVI) (3). MVI represents a critical biomarker for stratifying postoperative recurrence risk and guiding individualized treatment decisions (4). Currently, MVI status is determined primarily by postoperative immunohistochemical analysis, which—owing to its invasive nature and single-site sampling limitation—fails to comprehensively capture intratumoral spatial heterogeneity and precludes preoperative dynamic monitoring (5, 6).

Radiomics has emerged as a promising approach for noninvasive assessment of HCC by enabling high-throughput extraction of quantitative imaging features from routine medical images (7). Compared with conventional imaging interpretation, radiomics-based models can improve the objectivity and accuracy of prognostic prediction (8). However, most existing models rely on whole-tumor characteristics and do not account for localized phenotypic variations within the tumor such as vascular distribution patterns or metabolically active subregions thereby limiting their ability to characterize the spatial heterogeneity underlying MVI.

Habitat analysis addresses this limitation by applying unsupervised clustering algorithms (e.g., k-means) to partition tumors into subregions with similar biological properties, such as hypervascular zones and necrotic cores, and quantifying their textural features and spatial topological relationships (9, 10). This strategy allows precise characterization of intratumoral heterogeneity (11). In this study, we innovatively integrated habitat analysis with radiomics to develop a preoperative, noninvasive model for predicting MVI status in HCC. The purpose was to overcome the biological characterization bottleneck inherent in conventional approaches and to provide a quantifiable and interpretable imaging biomarker for clinical use.

## Materials and methods

### Patients

This retrospective study was approved by the Institutional Review Board of our hospital (Approval No.: QT2025045). Because of the retrospective design, the requirement for written informed consent was waived. Between January 2022 and January 2025, a total of 367 patients who underwent surgical resection with pathologically confirmed hepatocellular carcinoma (HCC) were identified from our institutional database.

Inclusion criteria were as follows: (1) age ≥ 18 years; (2) liver function classified as Child–Pugh class A or B; (3) pathologically confirmed HCC with definitive microvascular invasion (MVI) status and Edmondson grade; (4) contrast-enhanced CT performed within 1 week before surgery; (5) no prior history of antitumor therapy; and (6) if multiple lesions were present, the largest lesion consistent with both pathology and immunohistochemical findings was selected for analysis.

**Figure 1:**
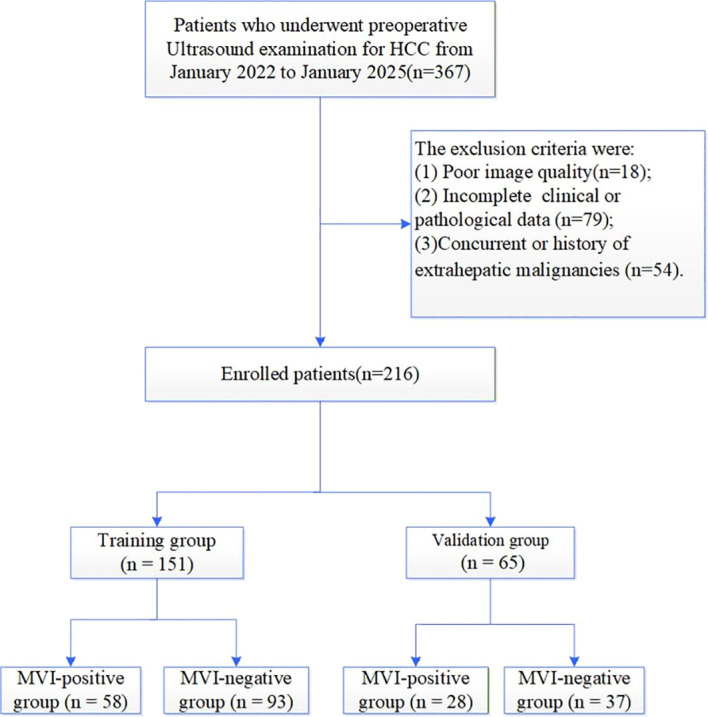
Flowchart of participant enrollment.

Exclusion criteria included: (1) poor image quality due to severe artifacts or incomplete imaging series (n = 18); (2) incomplete clinical or pathologic data (n = 79); and (3) history of or current malignancy other than HCC (n = 54). After excluding 151 ineligible patients, 216 patients were ultimately enrolled (**Figure 1**).

The cohort was divided into a training set and a validation set at a 7:3 ratio for model development and internal validation, respectively, comprising 151 patients (training set) and 65 patients (validation set).

### Imaging protocol

All patients underwent noncontrast and contrast-enhanced CT examinations using either a Siemens SOMATOM Definition AS 128 or a Siemens SOMATOM Definition AS 40 scanner (Siemens Healthineers, Forchheim, Germany). Scan parameters are detailed in the **Supplementary Information** (SI).

### Baseline evaluation

Demographic, tumor, and clinicopathologic data were collected, including age, sex, Edmondson grade, Ki−67 expression, CD10 expression, CD34 expression, hepatitis B virus infection status, serum α−fetoprotein (AFP) level, total bilirubin (TBIL), albumin (ALB), and prothrombin time (PT).

### Radiological image analysis

Radiological characteristics evaluated included maximum tumor diameter, presence of a capsule, tumor margin definition, and intratumoral necrosis. All CT images were initially reviewed independently by one abdominal radiologist with 8 years of experience and subsequently verified and classified by a second abdominal radiologist with 15 years of experience. In cases of disagreement, a third radiologist with 25 years of experience was consulted 8until consensus was reached for final classification. Throughout the evaluation, all three radiologists were blinded to patients’ clinical and pathologic information to ensure objectivity and independence.

### Pathological examination

Microvascular invasion (MVI) status was determined by two pathologists, each with more than 8 years of diagnostic experience. Specimens were obtained from regions suspicious on imaging (e.g., irregular tumor margins, areas of capsular breakthrough) and from portal vein branches within 1 cm of the tumor periphery. At least 5–8 tumor tissue blocks were sampled per patient, including tumor–normal tissue interfaces. MVI-negative cases were defined as M0; MVI-positive cases were classified as M1 or M2.

### Tumor segmentation and extraction of radiomic features

Preoperative CT images were exported in Digital Imaging and Communications in Medicine (DICOM) format and imported into the open-source software ITK−SNAP (version 3.8; http://www.itksnap.org/). To eliminate the effect of varying slice thicknesses on feature stability, we resampled all DICOM images to isotropic 1×1×1 voxels. To reduce the dynamic range of grey-scale values and eliminate grey-scale variations between different devices, we performed Z-score normalization on the voxel intensities within the volume of interest (VOI). On each axial slice, a trained operator manually delineated the entire tumor contour to generate a VOI. If multiple lesions were present, only the largest lesion was analyzed. VOI segmentation was performed independently by two abdominal radiologists with 5 and 13 years of experience, respectively. Interobserver reproducibility was assessed using the intraclass correlation coefficient (ICC); an ICC > 0.8 was considered indicative of good consistency.

Radiomic features were extracted from the portal venous phase CT images using the open-source PyRadiomics platform (version 3.0.1). A total of 1,315 original features were computed, encompassing: first−order statistics; texture features including gray−level co−occurrence matrix (GLCM) and gray−level run−length matrix (GLRLM) descriptors; and higher−order features including wavelet−filtered features and morphological features.

### Habitat generation

For habitat subregion identification, local features were extracted for each voxel within the VOI, including local entropy (quantifying neighborhood intensity randomness) and local energy (reflecting intensity variation strength). These feature vectors characterized voxel attributes such as textural complexity and metabolic heterogeneity, providing a basis for biologically informed tumor subtyping.

The optimal number of clusters was determined by evaluating the Calinski–Harabasz (CH) index across cluster numbers ranging from 2 to 10, with k = 3 yielding the best within−cluster compactness and between−cluster separation. Each HCC lesion was thus partitioned into three functionally heterogeneous subregions using the k−means clustering algorithm. A total of 1,315 features were computed for each habitat, resulting in 3,945 radiomic features per lesion (1,315 × 3).

Minimum redundancy maximum relevance (mRMR) was applied to select the top 30 features for each modality. Feature importance was ranked based on out−of−bag (OOB) error from a random forest model; redundant features were removed after sorting by descending importance, and the thirty highest−ranking features were retained to construct the habitat analysis model. The model output a risk score representing the habitat−derived risk value.

### Feature selection and model construction

In this retrospective study of 216 patients, radiomic features were preprocessed prior to analysis. Continuous variables were normalized to the [0,1] range using min−max scaling, and outliers were addressed by median imputation. For clinical variables, backward stepwise regression was applied for dimensionality reduction to enhance interpretability and reduce overfitting risk.

Key radiomic features were selected using a random forest method. From the initial 1,315 raw features, mRMR was used to retain the top 30 features per modality. Feature importance was assessed via OOB error from the random forest; after ranking by descending importance and removing redundancy, the three most important features were selected to build the radiomics model. The cohort was randomly split into a training set (n = 151) and a validation set (n = 65) at a 7:3 ratio. Shapley additive explanation (SHAP) values were used to visualize the contribution of selected features. The model generated a radiomics risk score (Rad Score) as its final output.

**Figure 2:**
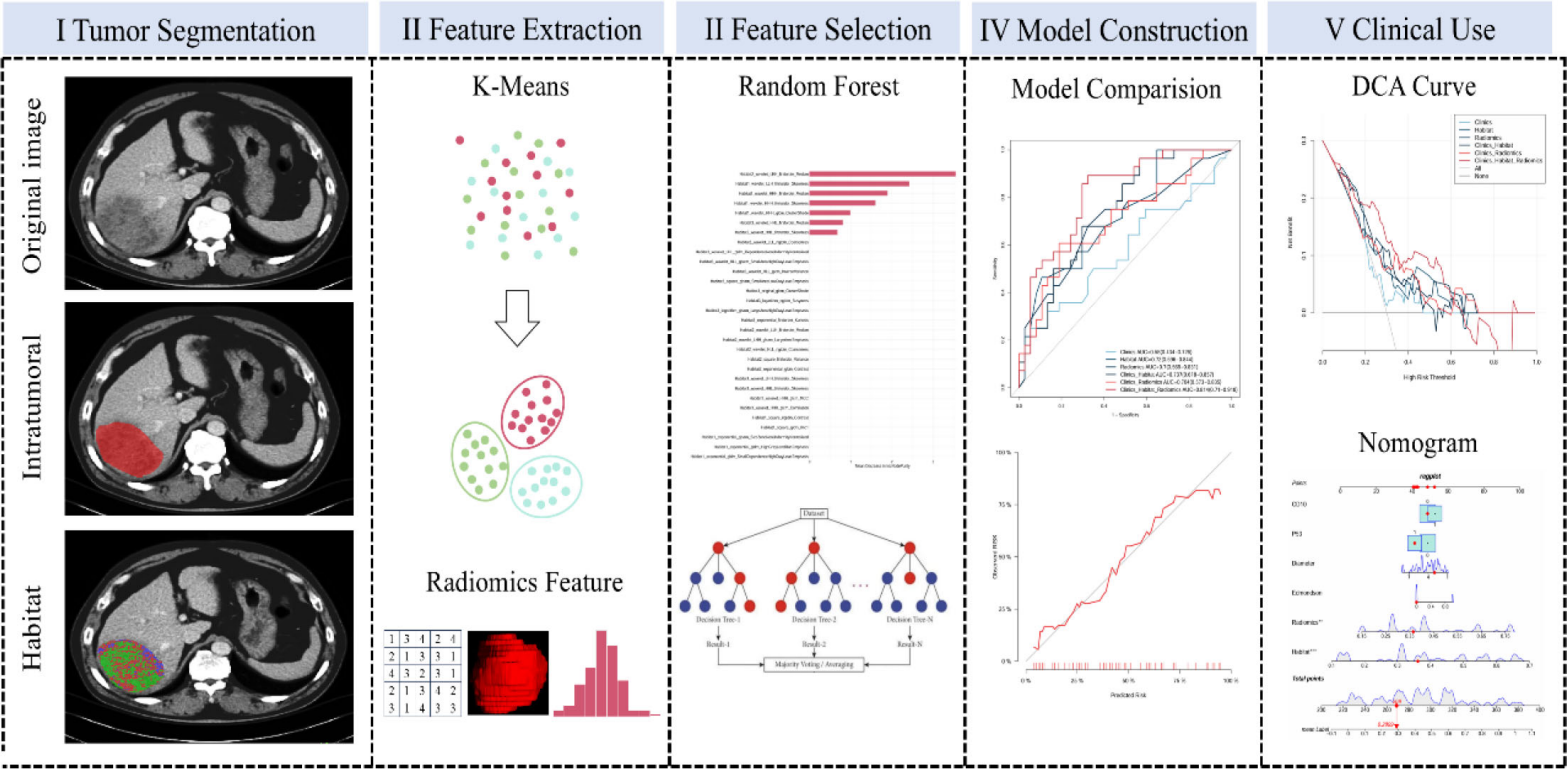
Overall workflow of this study.

The habitat risk score and radiomics score were then combined with clinically significant variables identified by logistic regression to construct an integrated predictive model, visualized as a nomogram. Correlations among variables in the combined model were also analyzed. The workflow of model construction is illustrated in **Figure 2**.

### Statistical analysis

All statistical analyzes were performed using R software (version 4.4.2; https://www.r-project.org). Continuous variables were compared using the Student t-test or the Mann–Whitney Utest, depending on the distribution of the data. Categorical variables were compared using the χ² test or Fisher exact test, as appropriate. Model performance was assessed by receiver operating characteristic (ROC) curve analysis. For each ROC curve, the area under the curve (AUC), accuracy, sensitivity, and specificity were calculated. Decision curve analysis (DCA) was used to evaluate clinical utility across a range of threshold probabilities. The DeLong test was applied to compare AUC values between different models. A two-sided Pvalue of less than 0.05 was considered statistically significant.

## Results

### Patient characteristics

A total of 216 patients (median age, 61.1 years; age range, 24–90 years) were included, comprising 86 patients with MVI-positive status and 130 with MVI-negative status. The cohort consisted of 22 women (10.2%) and 194 men (89.8%). Patients were divided into a Training Set (n = 151) and a Validation Set (n = 65).

The Training Set included 151 patients [median age, 61.0 years; 13 women (8.6%) and 138 men (91.4%)]. The Validation Set comprised 65 patients [median age, 61.5 years; 9 women (13.8%) and 56 men (86.2%)]. Detailed baseline characteristics of the Training and Validation Sets are summarized in **Table 1**. There were no significant differences between the two sets in terms of clinical or radiologic characteristics (*P >*0.05).

**Table 1:** .

Characteristics	Overall	Training Group	Validation Group	*P*-Value
n	216	151	65	
Gender, n (%)				0.326
0	22 (10.2)	13 (8.6)	9 (13.8)	
1	194 (89.8)	138 (91.4)	56 (86.2)	
Age, mean (SD)	61.1 (11.7)	61.0 (11.4)	61.5 (12.6)	0.776
Edmondson, n (%)				0.425
I-II	151(69.9)	108(71.5)	43(66.2)	
III-IV	65(30.1)	43(28.5)	22(33.8)	
P53, n (%)				1.000
0	122 (56.5)	85 (56.3)	37 (56.9)	
1	94 (43.5)	66 (43.7)	28 (43.1)	
CD10, n (%)				0.459
0	113 (52.3)	76 (50.3)	37 (56.9)	
1	103 (47.7)	75 (49.7)	28 (43.1)	
CD34, n (%)				0.625
0	63 (29.2)	46 (30.5)	17 (26.2)	
1	153 (70.8)	105 (69.5)	48 (73.8)	
Diameter, mean(SD)	4.0(2.0)	4.0(2.0)	4.1 (1.8)	0.804
Liver_cirrhosis, n (%)				0.227
0	128 (59.3)	85 (56.3)	43 (66.2)	
1	88 (40.7)	66 (43.7)	22 (33.8)	
HBsAg, n (%)				0.327
0	60 (27.8)	39 (25.8)	21 (32.3)	
1	156 (72.2)	112 (74.2)	44 (67.7)	
Capsule_appearance, n (%)				1.000
0	156 (72.2)	109 (72.2)	47 (72.3)	
1	60 (27.8)	42 (27.8)	18 (27.7)	
Tumor_margin, n (%)				0.881
0	131 (60.6)	91 (60.3)	40 (61.5)	
1	85 (39.4)	60 (39.7)	25 (38.5)	
Intratumoral_necrosis,,n (%)				0.526
0	69 (31.9)	46 (30.5)	23 (35.4)	
1	147 (68.1)	105 (69.5)	42 (64.6)	
AFP, n (%)				1.000
0	108 (50.0)	76 (50.3)	32 (49.2)	
1	108 (50.0)	75 (49.7)	33 (50.8)	
TB, mean (SD)	18.9 (12.3)	19.0 (10.5)	18.6 (15.9)	0.886
ALB, mean (SD)	38.5 (5.8)	38.6 (6.4)	38.2 (4.4)	0.657
PT, mean (SD)	12.3 (1.2)	12.4 (1.3)	12.2 (1.2)	0.358

### Statistical analysis of clinical features

A total of 16 clinical variables were collected and subjected to logistic regression for feature selection. Four variables demonstrating significant associations with MVI were identified: Edmondson grade, p53 expression, CD10 expression, and tumor diameter. A clinical risk score was calculated using logistic regression analysis, and a clinical model was subsequently constructed.

### Model construction for prediction of MVI

**Figure 3:**
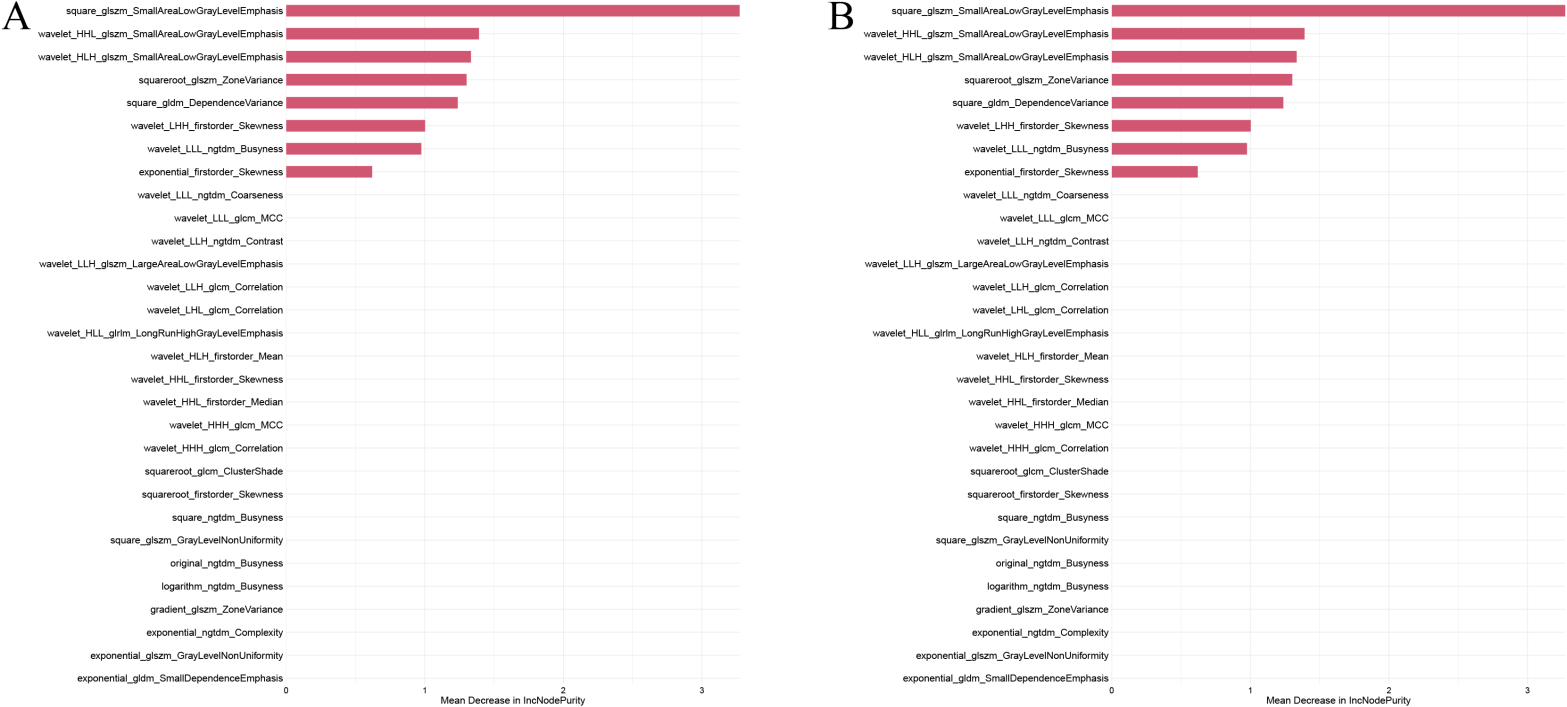
CT radiomics features and weight maps for each feature. **(A)** Habitat features. **(B)** Radiomic features.

From the 3,945 habitat features identified in the previous step, minimum redundancy maximum relevance (mRMR) was applied to retain 30 features. Feature importance was ranked based on out-of-bag (OOB) error from a random forest model; after sorting by descending importance and removing redundant features, the three highest-ranked features were selected to construct the habitat model (**Figure 3**).

Six predictive models were developed: (1) clinical model; (2) habitat model; (3) radiomics model; (4) clinical + habitat model; (5) clinical + radiomics model; and (6) integrated model combining habitat, radiomics, and clinical features.

### Model evaluation and visualization

**Figure 4:**
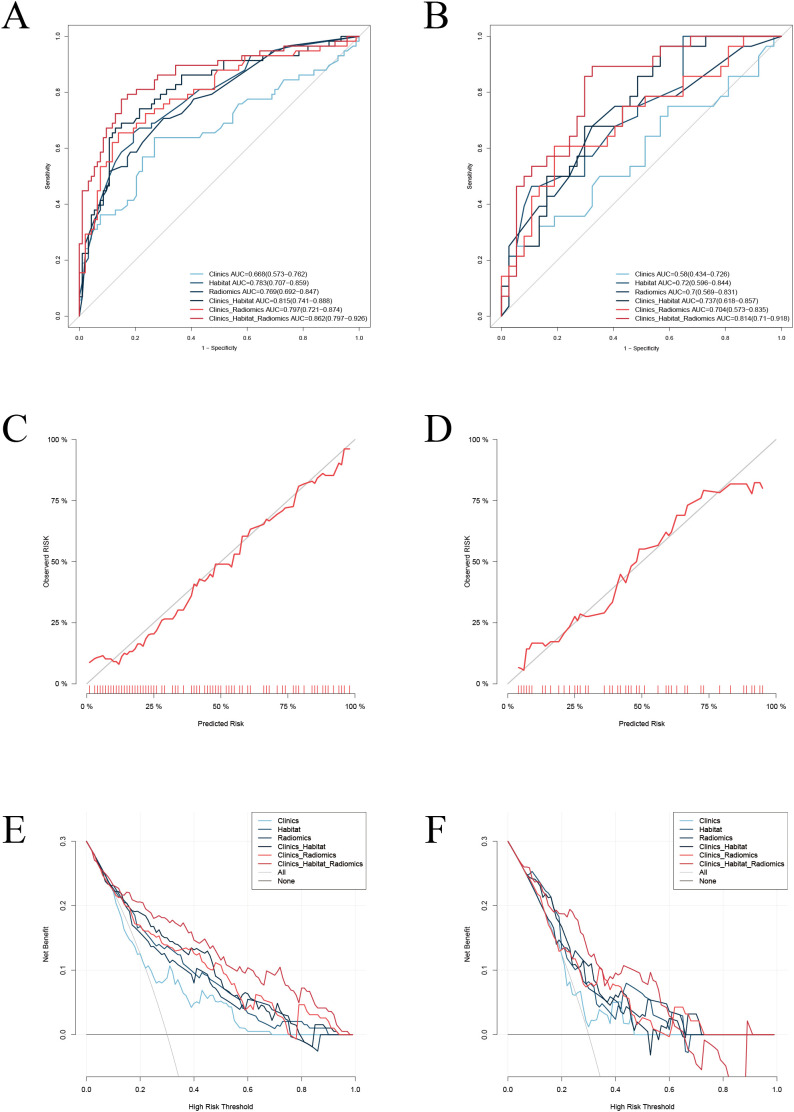
ROC, calibration, and DCA curves for all models. Participants were divided into training **(A, C, E)** and validation **(B, D, F)** cohort. **(A, B)** ROC curves: the ROC curves demonstrated the predictive performance of all models. The integrated model achieved the highest AUC values. **(C, D)** Calibration curves: the calibration curves showed good agreement between the predicted probabilities and the observed outcomes in both the training and validation cohorts, indicating that the integrated model was well-calibrated. **(E, F)** DCA, the DCA curves revealed that the integrated model provided significant clinical benefits within a specific range of threshold probabilities.

Receiver operating characteristic (ROC) curve analysis was performed to evaluate the performance of the six predictive models. The habitat model achieved an area under the ROC curve (AUC) of 0.783 (95% confidence interval [CI]: 0.707–0.859) in the training set and 0.720 (95% CI: 0.596–0.844) in the validation set. Among all models, the integrated habitat–radiomics–clinical model exhibited the best predictive performance (**Figures 4A, B**). Its AUC values were 0.862 (95% CI: 0.797–0.926) in the training set and 0.814 (95% CI: 0.710–0.918) in the validation set.

**Figure 5:**
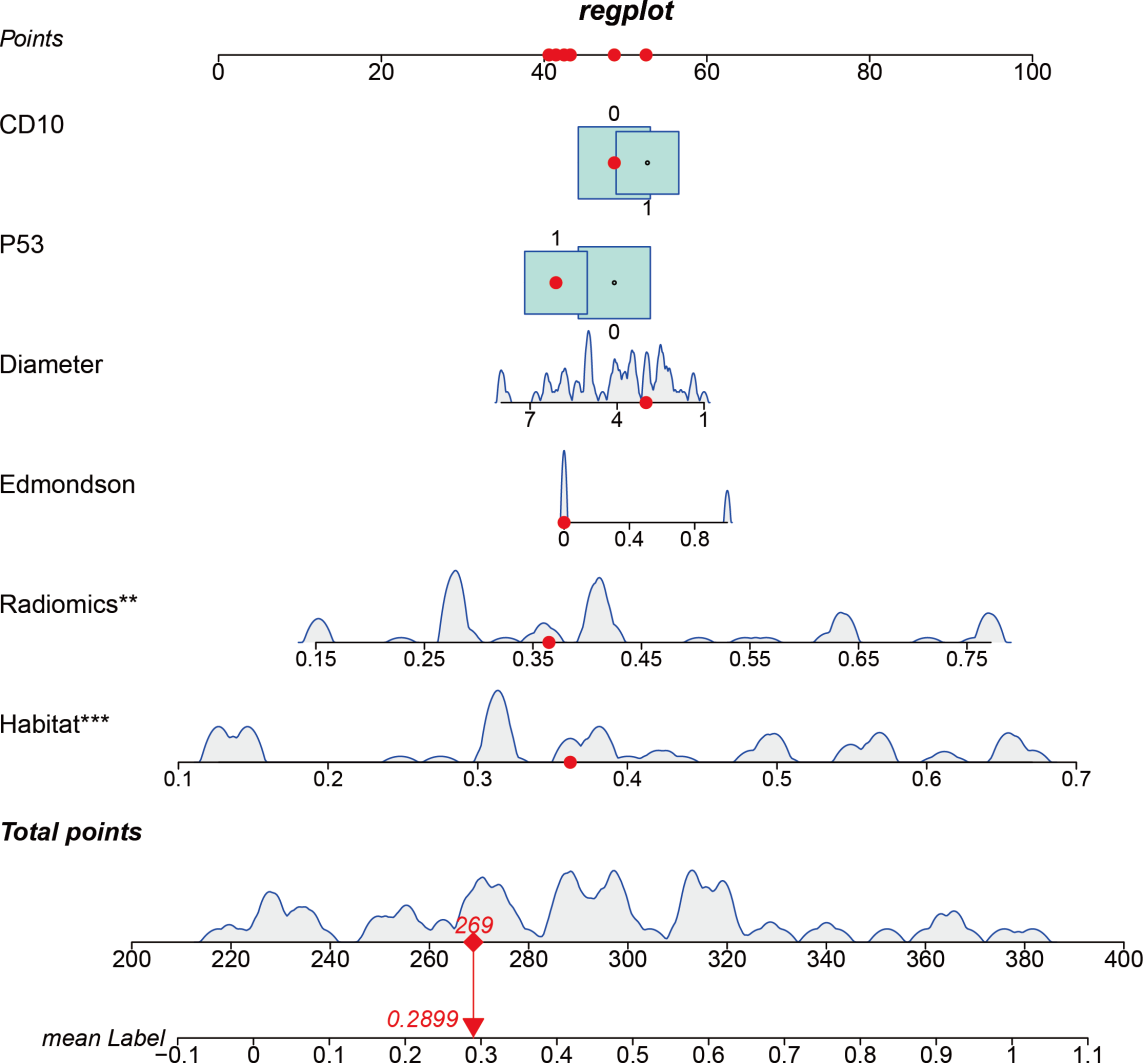
Visualization of the integrated model and analysis of variable correlations.

Based on these results, a nomogram was developed to visually represent the integrated model (**Figure 5**). Calibration performance was assessed quantitatively. The Hosmer–Lemeshow (H–L) goodness-of-fit test showed nonsignificant results in both the training cohort (*P* = 0.55) and the validation cohort (*P* = 0.60), indicating no significant deviation between observed outcomes and predicted probabilities. Calibration curves further demonstrated close agreement between predicted and actual event rates across all risk strata in the training set, with comparable calibration accuracy observed in the validation set (**Figures 4C, D**).

Decision curve analysis (DCA) revealed that, within a threshold probability range of 15%–65%, the integrated model yielded greater net benefit than the clinical model or the radiomics model alone (**Figures 4E, F**). The discriminative performance of the integrated model was significantly superior to that of each single-modality model. DeLong’s test showed that the AUC of the integrated model was significantly higher than that of the radiomics model (Z= –2.05, *P* < 0.05), the clinical model (Z= –3.21, *P* < 0.05), and the habitat model (|Z| > 1.96, *P* < 0.05), indicating a statistically significant advantage in distinguishing between MVI−positive and MVI−negative patients.

For visual representation of the integrated model and exploration of intervariable associations, we devised a nomogram that incorporates CD10, P53, Diameter, Edmondson grade, and radiomics features. This nomogram predicts the probability of MVI−positive status in HCC patients. The combination of clinicopathologic and radiomics parameters provides a concise framework for individualized preoperative risk estimation.

## Discussion

In this study, a multimodal model integrating CT-based habitat analysis, radiomics features, and clinicopathologic indicators significantly improved preoperative prediction of microvascular invasion (MVI) in hepatocellular carcinoma (HCC). The model achieved an area under the receiver operating characteristic curve (AUC) of 0.862 (95% CI: 0.797–0.926) in the training set and 0.814 (95% CI: 0.710–0.918) in the validation set, with excellent calibration (Hosmer–Lemeshow test, P = 0.60) and substantial clinical net benefit [decision curve analysis (DCA) showing a net benefit of 27% at a threshold probability of 30%]. These metrics outperformed both the clinical model and single-modality radiomics model. The integrated model was visualized as a nomogram (**Figure 5**), enabling individualized quantitative estimation of MVI probability and providing an intuitive decision aid for preoperative interventions such as screening for neoadjuvant transarterial chemoembolization (TACE).

Previous studies have reported that radiomic features extracted from the portal venous phase yield more stable MVI prediction than arterial phase features (12). Consistent with this, we derived both radiomic and habitat features from the CT portal venous phase. The habitat risk score and radiomics score (Radscore) each achieved AUC > 0.7 in the training, internal validation, and external validation cohorts, indicating robust diagnostic performance comparable to earlier findings (13).

By incorporating clinical-pathologic parameters (Edmondson grade, p53 and CD10 expression) with radiomics signatures (Radscore) and multimodal data including tumor diameter and habitat risk score, our integrated model markedly enhanced preoperative MVI prediction. Prior evidence has established that increasing tumor diameter correlates significantly with advanced clinical stage (P <0.05) and serves as an independent risk factor for MVI positivity (14). In the validation cohort, the integrated model achieved an AUC of 0.814(0.710-0.918), significantly outperforming the radiomics-only model (AUC=0.700, *P* = 0.03) and the clinical indicator model (AUC=0.580, *P* = 0.001), consistent with reported performance gains from multimodal fusion strategies. These results suggest that combining biologic characteristics with quantitative imaging information enables synergistic optimization of diagnostic performance, offering more reliable support for noninvasive MVI risk assessment in HCC.

The habitat-integrated model demonstrated superior discriminatory capability compared with whole-tumor radiomics and clinical models (AUC: 0.81 vs 0.70 vs 0.58). The core advantage of habitat radiomics lies in its capacity to capture intratumoral heterogeneity: using k-means clustering, we partitioned HCC lesions into three spatially contiguous subregions (Dice coefficient ≥ 0.85) with similar biologic behavior. Quantitative features were then extracted from each subregion independently. This approach mitigates feature bias introduced by including necrotic or benign components in whole-tumor analysis, thereby improving representation of malignant biologic properties. In the final habitat model, the wavelet texture feature Habitat2_wavelet_LHH_firstorder_Medianreceived the highest weight. Derived from three-level wavelet decomposition, this feature combines low-frequency (L) components preserving gross tumor architecture with high-frequency (HH) components capturing edge and microstructural details, and computes the median intensity of the LHH subband. Prior studies have shown that this feature sensitively reflects tumor metabolic heterogeneity and correlates with Wnt/β-catenin pathway activity and HIF-1α expression in non–small cell lung cancer (15), suggesting cross-cancer biologic relevance and potential as a noninvasive imaging biomarker for HCC aggressiveness.

Calibration was excellent (Hosmer–Lemeshow test, *P* = 0.62), and DCA revealed that the integrated model conferred significantly higher net benefit than the other six models (12.7% increase when threshold probability exceeded 20%). Based on risk stratification, patients with high MVI probability (>80%) may benefit from neoadjuvant TACE combined with targeted therapy to optimize preoperative planning, whereas those with low probability (<30%) could avoid overtreatment (16). DCA further indicated that when the threshold probability exceeded 20%, the integrated model substantially increased clinical net benefit; for such patients, postoperative follow-up intervals could be shortened to 3 months with multiphase contrast-enhanced CT surveillance for micrometastases (17).

Our study has several limitations. As a single-center retrospective cohort, it may be subject to selection bias; future multicenter prospective studies are needed to validate generalizability. CT texture features are susceptible to scanning parameter variability, and compatibility with different vendor platforms and low-dose protocols requires further investigation. Additionally, the model relies on preoperative static CT and does not capture habitat evolution after neoadjuvant therapy.

## Conclusions

The integration of tumor habitat analysis and radiomics overcomes traditional bottlenecks in MVI prediction, delivering high discrimination (AUC = 0.814, 95% CI: 0.710–0.918) and clinical net benefit as a noninvasive tool for preoperative staging of HCC. Future exploration of molecular mechanisms, such as linking imaging signatures to β-catenin pathway activity, and development of real-time dynamic models may establish this strategy as an imaging biomarker to guide individualized treatment decisions.

## Data Availability

The original contributions presented in the study are included in the article/**Supplementary Material**. Further inquiries can be directed to the corresponding authors.
