# Supplementary material for: CT-based habitat imaging integrated with radiomics and clinicopathology for noninvasive prediction of microvascular invasion in hepatocellular carcinoma
Source: Front Oncol. 2026 May 22;16:1832615. doi: 10.3389/fonc.2026.1832615 (PMC13236552; doi:10.3389/fonc.2026.1832615)
Supplement: Supplementary file 1 [file Table1.docx]

**Supplementary Materials**

**Supplementary Data S1** Detailed parameters of CT scanning

The scanning parameters were as follows: tube voltage of 120 kVp with automatic tube current modulation; detector collimation of 128×0.625 mm or 40×0.625 mm; matrix size of 512×512; slice thickness of 5 mm; and reconstruction interval of 2 mm. All patients underwent non-contrast abdominal scanning, as well as contrast-enhanced CT scans during the arterial phase (AP) and portal venous phase (VP).

The contrast-enhanced CT acquisition protocol was as follows: Patients were instructed to fast for 6 hours before scanning and orally administered 800–1000 ml of a negative contrast agent to distend the gastrointestinal tract. Iodinated contrast medium (Ultravist 370, 1.5 ml/kg) was injected via the antecubital vein at a rate of 3.0–3.5 ml/s using a Medrad Vistron CT power injector (Medrad Inc., USA). The arterial phase scan was initiated 35 seconds after contrast injection, and the portal venous phase scan was initiated 70 seconds after injection. The acquired images were subsequently transferred to a picture archiving and communication system (PACS) workstation for analysis.

TABLE 1 Detailed parameters of CT scanning

| Parameters | Siemens SOMATOM Definition AS 128 | Siemens SOMATOM Definition AS 40 |
| --- | --- | --- |
| Tube voltage (kVp) | 120 | 120 |
| Tube current (mA) | Auto | Auto |
| Rotation times (s) | 0.5 | 0.5 |
| Field of view (mm) | 300-450 | 300-500 |
| Detector collimation (mm) | 128×0.625 | 40×0.625 |
| Pixel size | 512×512 | 512×512 |
| Slice thickness (mm) | 5 | 5 |

| **Supplementary Data S2.** Habitat and Radiomics features retained after Mrmr screening |
| --- |
| \| Habitat Features \| \| --- \| \| 1. Habitat2_wavelet_HHL firstorder Skewness  2. Habitat3_wavelet_HLL_glcm_InverseVariance  3. Habitat3_exponential_firstorder Kurtosis  4. Habitat1_wavelet HHH_glcm_Correlation  5. Habitat3_square_glszm_SmallAreaLowGrayLevelEmphasis  6. Habitat2_square firstorder Variance  7. Habitat1_exponential_gldm_SmallDependenceHighGrayLevelEmphasis  8. Habitat2_wavelet HLL_ngtdm_Coarseness  9. Habitat3_original_glcm_ClusterShade  10. Habitat3_wavelet_HHL firstorder Median  11. Habitat3_ wavelet LHL_gldm_DependenceNonUniformityNormalized  12. Habitat3_wavelet HLL_glszm_SmallAreaHighGrayLevelEmphasis  13. Habitat1_square_glcm_lmc1  14. Habitat1_wavelet_HHH_glcm_ClusterShade  15. Habitat1_wavelet_LHH_firstorder_Skewness  16. Habitat2_exponential_glcm_Contrast  17. Habitat3_logarithm_ngtdm_Busyness  18. Habitat1_wavelet_HHH_firstorder_Median  19. Habitat1_exponential_glrlm_HighGrayLevelRunEmphasis  20. Habitat2_wavelet LHH_glszm_LargeAreaEmphasis \| \| 21. Habitat3_logarithm_glszm_LargeAreaHighGrayLevelEmphasis \| \| 22. Habitat1_wavelet_HHL_firstorder_Skewness \| \| 23. Habitat2_wavelet_LLH_firstorder_Median \| \| 24. Habitat1_wavelet_LLH firstorder_Skewness \| \| 25. Habitat3_wavelet_LLL_ngtdm_Coarseness \| \| 26. Habitat2_wavelet_LHH_firstorder_Median \| \| 27. Habitat1_square_ngtdm_Contrast \| \| 28. Habitat1_exponential_glszm_SizeZoneNonUniformityNormalized \| \| 29. Habitat1_wavelet_HHH_glcm_MCC \| \| 30. Habitat1_wavelet_HHH_firstorder_Skewness \| |

| Radiomics Features |
| --- |
| 1. wavelet_LLH_ngtdm_Contrast  2. wavelet HHH_glcm MCC  3. wavelet HLH_glszm_SmallAreaLowGrayLevelEmphasis  4. wavelet LHL_ glcm_Correlation  5. logarithm_ngtdm_Busyness  6. wavelet_LLL_glcm_MCC  7. squareroot_glszm_ZonevVariance  8. exponential_firststorder_Skewness  9. wavelet_HLH_firstorder_Mean  10. wavelet LLL_ngtdm_Busyness  11. exponential_glszm_GrayLevelNonUniformity  12. original_ngtdm_Busyness  13. squareroot_glcm_ ClusterShade  14. wavelet_HHL_firstorder_Median  15. exponential_ngtdm_Complexity  16. square_gldm_DependenceVariance  17. wavelet_LHH_firstorder_Skewness  18. gradient_glszm_ZoneVariance  19. wavelet_LLH_glszm_LargeAreaLowGrayLevelemphasis  20. wavelet_LLH_glcm_Correlation |
| 21. wavelet_HLL_glrlm_LongRunHighGrayLevelemphasis |
| 22. square_glszm_GrayLevelNonUniformity |
| 23. square_ngtdm_Busyness |
| 24. wavelet_LLL_ngtdm_Coarseness |
| 25. squareroot_firstorder_Skewness |
| 26. exponential_gldm_SmallDependenceEmphasis |
| 27. square_glszm_SmallAreaLowGrayLevelEmphasis |
| 28. wavelet_HHL_firstorder_Skewness |
| 29. wavelet_HHL_glszm_SmallAreaLowGrayLevelEmphasis |
| 30. wavelet_HHH_glcm_Correlation |

**Supplementary Data S3.** **The formulae of the logistic regression model in predictive models**

The formula of all model using Logistic model was as following:

（A) clinical risk score (CRS) was computed using the linear predictor of the final model: CRS = -2.06+ 0.59 × Edmondson + 0.61 × P53 + 0.62 × CD10 + 0.20 × Diameter

(B) Clinics_Radiomics model risk score(CRRS): CRRS = -3.86 + 0.56 × Edmondson + 0.63 × P53 + 0.69 × CD10 + 0.08 × Diameter + 5.48 × Radiomics

(C) Clinics_Habitat model risk score(CHRS): CHRS = -4.66 + 0.31 × Edmondson + 0.57 × P53 + 0.50 × CD10 + 0.21 × Diameter + 6.67 × Habitat

(D) Integrated model risk score(IRS):IRS = -5.88 + 0.34 × Edmondson + 0.53 × P53 + 0.54 × CD10 + 0.09 × Diameter + 6.06 × Habitat + 4.70 × Radiomics

**Supplementary Data S4.** Model performance of each cohort

| Model | Model | AUC (95% CI) | ACC | TPR | TNR | PPV | NPV | BER |
| --- | --- | --- | --- | --- | --- | --- | --- | --- |
| Training cohort | Clinics | 0.668(0.573-0.762) | 0.609 | 0.638 | 0.591 | 0.493 | 0.724 | 0.385 |
|  | Habitat | 0.783(0.707-0.859) | 0.715 | 0.690 | 0.731 | 0.615 | 0.791 | 0.290 |
|  | Radiomics | 0.769(0.692-0.847) | 0.689 | 0.707 | 0.677 | 0.577 | 0.788 | 0.308 |
|  | Clinics_Habitat | 0.815(0.741-0.888) | 0.728 | 0.793 | 0.688 | 0.613 | 0.842 | 0.259 |
|  | Clinics_Radiomics | 0.797(0.721-0.874) | 0.715 | 0.776 | 0.677 | 0.600 | 0.829 | 0.273 |
|  | Clinics_Habitat_Radiomics | 0.862(0.797-0.926) | 0.768 | 0.845 | 0.720 | 0.653 | 0.882 | 0.217 |
| Validation cohort | Clinics | 0.580(0.434-0.726) | 0.523 | 0.536 | 0.514 | 0.455 | 0.594 | 0.475 |
|  | Habitat | 0.720(0.596-0.844) | 0.631 | 0.607 | 0.649 | 0.567 | 0.686 | 0.372 |
|  | Radiomics | 0.700(0.569-0.831) | 0.662 | 0.750 | 0.595 | 0.583 | 0.759 | 0.328 |
|  | Clinics_Habitat | 0.737(0.618-0.857) | 0.677 | 0.679 | 0.676 | 0.613 | 0.735 | 0.323 |
|  | Clinics_Radiomics | 0.704(0.573-0.835) | 0.615 | 0.750 | 0.513 | 0.538 | 0.731 | 0.368 |
|  | Clinics_Habitat_Radiomics | 0.814(0.710-0.918) | 0.723 | 0.893 | 0.595 | 0.625 | 0.880 | 0.256 |
